# Characterization of aminoglycoside resistance in multidrug-resistant *Klebsiella pneumoniae* isolates

**DOI:** 10.1590/S1678-9946202567062

**Published:** 2025-10-03

**Authors:** Saidy Vásconez Noguera, Ana Paula Marchi, Marina Farrel Côrtes, Nazareno Scaccia, Roberta Cristina Ruedas Martins, Maura Salaroli de Oliveira, Flavia Rossi, Anna Sara Levin, Silvia Figueiredo Costa, Lauro Vieira Perdigão

**Affiliations:** 1 Universidade de São Paulo, Faculdade de Medicina, Instituto de Medicina Tropical de São Paulo, São Paulo, São Paulo, Brazil; 2 Pontificia Universidad Católica del Ecuador, Facultad de Medicina, Quito, Pichincha, Ecuador; 3 Universidade de São Paulo, Faculdade de Medicina, Departamento de Moléstias Infecciosas e Parasitárias, São Paulo, São Paulo, Brazil; 4 Centres for Antimicrobial Optimisation Network, São Paulo, São Paulo, Brazil; 5 Universidade de São Paulo, Faculdade de Medicina, Hospital das Clínicas, São Paulo, São Paulo, Brazil; 6 Universidade de São Paulo, Faculdade de Medicina, Hospital das Clínicas, Divisão de Laboratório Central Serviço de Microbiologia Clínica, São Paulo, São Paulo, Brazil

**Keywords:** Klebsiella pneumoniae, Multidrug-resistance, Aminoglycosides, Whole-genome sequencing, One Health

## Abstract

Multidrug-resistant (MDR) *Klebsiella pneumoniae*, particularly the lineages resistant to carbapenems and aminoglycosides, is an escalating global public health threat across human, animal, and environmental reservoirs. We examined phenotypic and genetic features of MDR *K. pneumoniae* isolates. A total of 70 *K. pneumoniae* strains were collected from clinical (n=55), environmental (n=7), and animal (n=8) sources. To better understand the evolutionary relationship between these isolates, a phylogenetic analysis was performed alongside 35 publicly available *K. pneumoniae* genomes from NCBI and Pathogenwatch. Whole-genome sequencing (WGS) revealed that 43 isolates carried the *bla*_KPC_ gene, including *bla*_KPC-2_ and *bla*_KPC-3_ variants, with different susceptibility profiles to aminoglycosides. Among all isolates, 84% (n = 59/70) were resistant to amikacin and 53% (n = 37/70) were resistant to gentamicin. Aminoglycoside resistance was primarily associated with aminoglycoside-modifying enzymes, including *aph(3’)-Ia* (52%), *aac*(3)-IIa/*aad*A2 (49%), and *aac(6’)-Ib-cr* (37%). Additionally, 16S rRNA methyltransferases *rmtB* and *rmtG* were detected in 14% of isolates and were associated with high-level amikacin MICs. Overall, 81% of strains were non-susceptible to at least one aminoglycoside, underscoring the clinical importance of these determinants. Phylogenetic analysis based on WGS data showed two main clusters (A and B), and the multilocus sequence type ST11 predominated among Brazilian isolates. Our findings showed a heterogeneous distribution of sequence type profiles across the two clusters and a close relationship between *K. pneumoniae* strains from human, animal, and environmental sources, highlighting the need for integrated One Health surveillance.

## INTRODUCTION

*Klebsiella* is a genus of opportunistic bacteria frequently linked to severe healthcare-associated infections. Since the early 2000s, *Klebsiella pneumoniae* is the most relevant member of Enterobacteriaceae family, especially due to its ability to acquire and spread multiple antibiotic resistance mechanisms, including resistance to aminoglycosides^1^. Moreover, resistance to beta-lactams and polymyxins in *K. pneumoniae* isolates from hospital settings has been increasing. *K. pneumoniae* commonly colonizes or infects critically ill patients, with mortality rates ranging from 40% to 70%. This high risk is also partly attributable to treatment limitations, such as the lack of new antibiotics^2^.

Antimicrobial overuse across multiple sectors has led to the release of large amounts of antibiotics into the environment, creating selective pressure for the development and acquisition of resistance genes by bacteria. Lepuschitz *et al*.^3^ demonstrated a link between environmental and clinical isolates of extended-spectrum β-lactamase (ESBL) and carbapenemase-producing *K. pneumoniae* by identifying identical clones in both hospital settings and a nearby river. Recent studies showed the importance of assessing health risks associated with antibiotic resistance linked to antibiotic residues, including 16S rRNA methylases and aminoglycoside-modifying enzymes^4^. At least one aminoglycoside resistance gene—mainly *aac(6’)-Ib* variants, which are known to reduce the efficacy of commonly used aminoglycosides—has been frequently detected^5^.

Currently, due to favorable *in vitro* susceptibility profiles, aminoglycoside-containing regimens are being considered as alternative treatment options. However, the increased use of aminoglycoside has led to the emergence of other resistance mechanisms, including 16S rRNA methylases and aminoglycosides-modifying enzymes. Most genes encoding 16S-RMTases are located on mobile genetic elements, such as plasmids, which often have a broad host range^6^. During the COVID-19 pandemic, there was a high prevalence of multidrug-resistant *Klebsiella pneumoniae* (MDR-Kp), with widespread resistance to aminoglycosides largely driven by the presence of 16S rRNA methyltransferase genes, particularly *rmtD*. This highlights the urgent need for ongoing surveillance and strengthened infection control measures^7^. According to the Brazilian Health Regulatory Agency (ANVISA – Agencia Nacional de Vigilancia Sanitaria), a microorganism is considered MDR when it exhibits non-susceptibility to at least one agent in three or more antimicrobial categories.

Understanding the mechanisms of aminoglycoside resistance and the factors contributing to its emergence and dissemination is critical toward developing effective control strategies. This includes identifying transmission routes, as evidenced by studies showing the co-occurrence of resistance determinants in clinical and environmental Enterobacteriaceae isolates, highlighting the role of mobile genetic elements in spreading MDR bacteria across diverse environments^8^.

This study aims to explore the distribution of aminoglycoside resistance genes and provide insights into the potential dissemination pathways of *K. pneumoniae* isolates from different sources.

## MATERIALS AND METHODS

### Ethics

The study was approved by the Ethics Committees of Hospital das Clinicas under reference Nº 5.283.703.

### Study design and bacterial strains

An initial phenotypic analysis was conducted on 35 clinical *K. pneumoniae* strains. These isolates were previously isolated from blood, urine, wound, tracheal aspirate, and abscess infections at Hospital das Clinicas, Faculdade de Medicina da Universidade de Sao Paulo (HCFMUSP), Brazil (Group 1; Supplementary Table S1). Genomic DNA was extracted with the QIAamp^®^ DNA Mini Kit (Qiagen, Hilden, Germany).

These isolates underwent whole-genome sequencing (WGS) using the MiSeq system (Illumina; San Diego, California, USA). Genome annotation was performed using Prokka (version 1.14.6, Monash University, Australia) and PATRIC (version 1.02, Pathosystems Resource Integration Center, Bacterial Bioinformatics Resource Center, USA). Sequence types (ST) and resistance genes were determined using MLSTfinder and ResFinder. Manual curation was conducted using the Artemis genome browser (version 17.1, Sanger Institute, UK) to inspect the genetic context of aminoglycoside resistance genes. PlasmidFinder was used to identify plasmid replicon types, and the location of resistance genes on contigs containing plasmid replicons was used to infer whether these genes were plasmid-borne. For genome assembly, *K. pneumoniae* MGH78578 (GenBank accession Nº CP000647.1) was used as the reference strain.

The sequence data produced in our study were deposited at DDBJ/ENA/GenBank under the BioProject accession Nº PRJNA377546 at the National Center for Biotechnology Information (NCBI). Individual genome accession numbers for each strain are listed in Supplementary Table S1.

To better understand the evolutionary relationship between the isolates, a phylogenetic analysis was performed with additional 35 publicly available *K. pneumoniae* genomes retrieved from NCBI and Pathogenwatch (Group 2; Supplementary Table S1). The genomes were first located in public databases, and the inclusion criteria were: documented MDR phenotype with reported susceptibility profiles for amikacin and gentamicin; detection of ≥ 1 aminoglycoside-modifying enzyme (AME) gene by ResFinder; and complete metadata for year, country, and source. Exclusion criteria were duplicated assemblies, metagenomic environmental records without metadata, or genomes lacking aminoglycoside susceptibility data. To avoid overrepresentation, a stratified random sampling approach limited selection to ≤ 12 genomes per source category, yielding 20 clinical human, eight animal, and seven environmental isolates. No more than two genomes per country-year were retained, and at least one isolate from each inhabited continent was included.

These publicly sourced genomes reflect both geographical and temporal diversity, aiming to provide a more comprehensive understanding of *K. pneumoniae* resistance to aminoglycosides. All genomes were analyzed as described in the following sections.

### Phenotypic characterization of clinical bacterial isolates

Group 1 genomes (35 clinical isolates from HCFMUSP; Supplementary Table S1) were analyzed for antibiotic susceptibility using *in vitro* laboratory methods, following the Clinical and Laboratory Standards Institute (CLSI) guidelines. Susceptibility to amikacin (AK), gentamicin (GEN), meropenem (MEM), and colistin (COL) was assessed using the Sensititre GNX3F system (TREK Diagnostic Systems, Cleveland, OH, USA) according to the manufacturer’s instructions to determine the resistance profile to these first-line antibiotics. For the remaining 35 isolates (Group 2), detailed information—including NCBI accession numbers—is provided in Supplementary Table S1.

### Resistance genes and phylogenetic analysis of K. pneumoniae genomes

Resistance genes and multilocus sequence typing (MLST) were evaluated using Resfinder and MLSTfinder, respectively^9^. Phylogenetic analysis was performed using the REALPHY tool (version 1.12, Centre for Genomic Epidemiology, Technical University of Denmark), based on single nucleotide polymorphisms (SNPs) from all 70 sequenced genomes, using default parameters. The tree was constructed with 500 bootstrap replications. Sequences were mapped with the reference genome *K. pneumoniae* MGH78578 (GenBank accession number CP000647.1) using Bowtie2 (version 2.0, Johns Hopkins University, Baltimore, MD, USA). All SNPs were manually verified and analyzed using Seaview (version 4.9, Laboratoire de Biométrie et Biologie Évolutive, CNRS, France) for both the multiple sequence alignment used in the phylogenetic analysis and the pairwise alignments obtained separately using Bowtie2. Additionally, a synteny analysis was performed using Mauve (version 2.4.0, Darling Lab, University of Wisconsin-Madison, USA) for isolates KP57 and KP04. Contigs for each sample were first reordered using *K. pneumoniae* MGH78578 as the reference genome. Subsequently, reordered contigs were aligned using Progressive Mauve.

## RESULTS

A total of 79% (n = 55/70) of strains were obtained from humans, 11% (n = 8/70) from animals, and 10% (n = 7/70) from environmental sources. Among human isolates, 73% (n = 40/55) were from Brazil, and of these, 53% (n = 21/40) were recovered from blood samples. The MIC range, MIC_50_, and MIC_90_ values for Group 1 are shown in Table 1.

**Table 1:** Antimicrobial resistance rates and MIC distribution of KP isolates (Group 1)

Antibiotic	MIC (μg/mL)			
	Range	MIC_**50**_	MIC_**90**_	Resistance (%)
Meropenem	1-≥ 16	8	16	88.6
Colistin	0.125-64	24	64	88.6
Amikacin	≤ 4-64	15	32	68.6
Gentamicin	≤ 0.5-> 256	64	> 256	40.0

MIC = minimum inhibitory concentrations; MIC50 = MIC at which 50% of the isolates tested are inhibited; MIC90 = MIC at which 90% of the isolates tested are inhibited; KP = *Klebsiella pneumoniae*.

**Figure 1:**
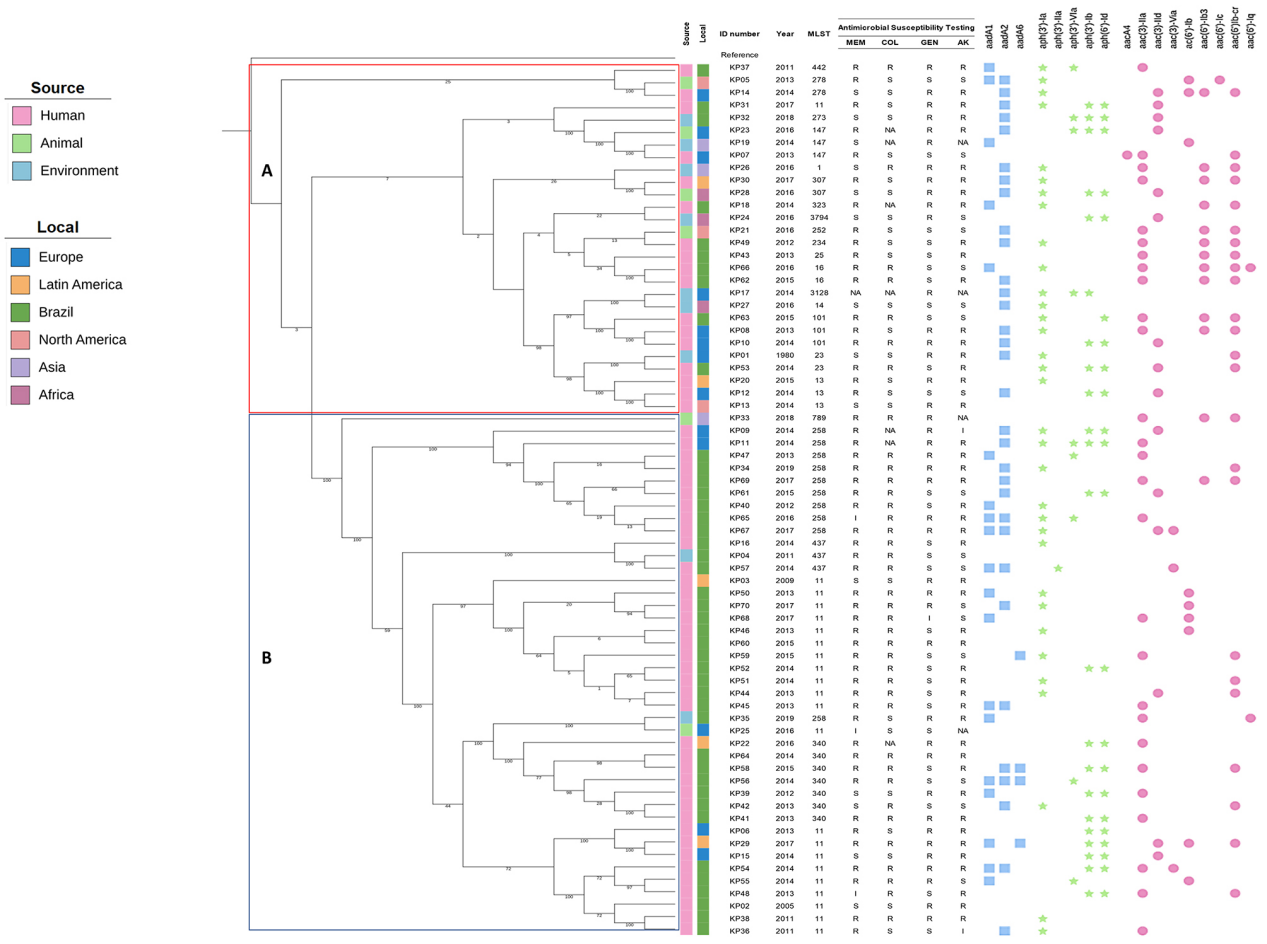
Phylogeny of 70 *K. pneumoniae* isolates. Tree analyses showed two main clusters (A and B). The first column shows the isolation source, the second column shows the isolation region, followed by the ID sample column, year of isolation, MLST, antimicrobial susceptibility testing, and aminoglycoside resistance genes. Blue square, green star, and pink circle show the adenyltransferase, phosphotranferase, and acetyltransferases genes, respectively.

Regarding genotypic characterization, MLST analyses showed that the 70 *K. pneumoniae* isolates belonged to 22 ST. ST11 was the most prevalent (n = 15), followed by ST258 (n = 8) and ST340 (n = 5) among Brazilian isolates. A total of 19,026 SNPs were analyzed in the phylogenetic analysis. Isolates were grouped into two major clusters (A and B), displaying a heterogeneous distribution regarding isolation source, geographic origin, and resistance profile (Figure 1). Interestingly, no correlation was observed between clustering and sequence type; for instance, isolates from ST11 were distributed across four different clusters with isolates from ST273, ST147, ST258, and ST340. Cluster A comprised 28 isolates from Latin America (including Brazil), North America, Europe, Asia, and Africa, belonging to 17 different STs. These isolates shared 17,260 SNPs and exhibited greater genetic diversity.

Cluster B comprised 42 genomes sharing 4,767 SNPs, and included most of the Brazilian isolates (79%). The phylogenetic analysis showed eight potential transmission events in which humans, animals, and/or environment samples were clustered. For these cases, a separate SNP analysis was performed to evaluate possible transmission. The following pairs shared SNP counts as indicated: KP05 (animal) and KP14 (human) shared 3,061 SNPs; KP19 (environment) and KP07 (human) shared 4,430 SNPs; KP30 (human) and KP28 (animal) shared 538 SNPs; KP18 (human) and KP24 (environment) shared 32,516 SNPs; KP21 (animal) and KP49 (human) shared 29,191 SNPs; KP01 (environment) and KP53 (human) shared 645 SNPs; KP04 (environment) and KP57 (human) shared 60 SNPs; KP35 (environment) and KP25 (animal) shared 2,301 SNPs.

Interestingly, most of these sample pairs originated from different locations, except for KP04 and KP57, which were both from Brazil (Figure 1).

**Figure 2:**
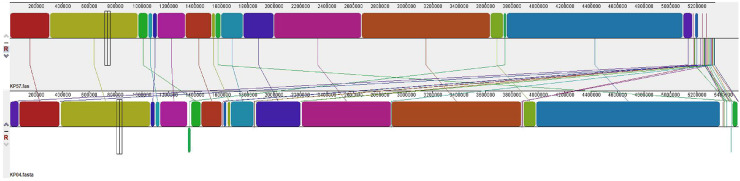
Synteny analysis of the pair KP04 (environment) and KP57 (human) using Mauve.

Visual genomic architecture analysis for KP04 and KP57 showed that these genomes share most of the genetic loci, with large syntenic blocks largely co-located (shown in colors). However, some differences were observed, including three genomic gaps (shown in white), several translocations, and one inversion affecting regions associated with mobile genetic elements and hypothetical proteins (Figure 2).

Cluster B comprises 42 isolates from Latin America (including Brazil), Europe, and Asia, belonging to different STs: ST11, ST258, ST340, ST437, and ST789, sharing 4,767 SNPs. Among Brazilian genomes (n = 35), ST11 accounted for 42.9% (15/35), followed by ST258 (22.9%, 8/35) and ST340 (14.3%, 5/35). Together, these three lineages represented 80% of the national collection, matching the dominant clones previously described in Brazilian surveillance studies^10^. Genotypic analysis showed that ST11 was the predominant lineage circulating in Brazilian isolates from 2011 to 2016, primarily originating from Hospital das Clinicas (HCFMUSP) and the Hospital of Londrina, Parana State. ST437 isolates from humans (rectal swab and perianal fluid) clustered with environmental samples (urban river) within Sao Paulo State, Brazil, over a period of less than three years. Moreover, ST258 was observed in a urine sample from a dog in Brazil in 2019, clustering with environmental sample (river) ST11 collected in 2016 from Austria. One human isolate from Colombia clustered with six human isolates from Brazil belonging to ST340 with 680 SNPs, from 2013 to 2016 (Figure 1).

Regarding phenotypic resistance profiles, 81% (n = 57/70) of isolates presented resistance to aminoglycosides, and 74% (n = 52/70) to meropenem, as determined by the broth microdilution method. Genotypic resistance analysis showed diverse range of extended-spectrum β-lactamase genes, including *bla*_KPC_, *bla*_TEM_, *bla*_SHV_, *bla*_CTX-M_, and *bla*_OXA_. Metallo-β-lactamase genes (*bla*_NDM1,5,7,9_) were detected in carbapenem-resistant strains from various sources. Notably, the predominant carbapenemase among these strains was *bla*_KPC_, followed by *bla*_TEM_, *bla*_SHV_, *bla*_CTX-M_, and *bla*_OXA_. Additionally, 93% (n = 65/70), 54% (n = 38/70), and 53% (n = 37/70) of isolates harbored AMEs, *bla*_KPC-2_, and *bla*_TEM_ genes, respectively (Supplementary Table S1). Aminoglycoside-resistant genes were observed in 65 samples (92.85%). The most frequent AMEs identified were *aph(3’)-Ia* (n = 34; 52.30%), *aadA2* and *aac(3)-IIa* (n = 32; 49.23%), *aac(6’)Ib-cr* (n = 24; 36.92%), *strA-strB* genes (n = 22; 33.84), *aadA1* (n = 19; 29.23%), *aac(3)-IId* and *aac(6’)-Ib* (n = 16; 24.61%), *aph(3’)-Via* (n = 13; 20.0%), *aadA6* (n = 4; 10.34%), *aac(3)-Via* (n = 3; 3.44%), *aac(6’)-Ib3*, *aac(6’)-Iq*, *aph(3’)-IIa*, and *aac(6’)-Ic* (n = 1; 3.44%).

Regarding plasmid content, several plasmid families were identified: IncFIB(K) (n = 68; 97.0%), IncFII(K) (n = 63; 90%), ColRNAI (n = 62; 88.5%), IncC (n = 56; 80.0%), IncN (n = 55; 78.5%), IncR (n = 50; 71.4%), IncU (n = 49; 70%), IncFIB (n = 48; 68.5%), IncQ1 (n = 45; 64.2%), InHI2 (n = 12; 17.14%), pKP1433 (n = 5; 7.1%). *In silico* comparison with *K. pneumoniae* plasmid pUR-KP0923 from Uruguay revealed a highly similar structure (> 99% similarity and 98% query coverage). This plasmid harbored the *aac (3’)-II* gene, and we also observed other components involved in transfer or transposition, hypothetical proteins, plasmid modification/maintenance functions, and other resistance genes (Supplementary Figure S1).

## DISCUSSION

The incidence of MDR *K. pneumoniae* infections has increased over the last decade, reflecting the extensive use of antimicrobial drugs. These findings directly contribute to this study, which explores the distribution of aminoglycoside-resistant genes and seeks to understand the dissemination pathways of *K. pneumoniae* isolates from different origins.

Among Brazilian isolates, ST11 was most frequently found in Group B, followed by ST258 and ST340. These STs have been previously reported as predominant clones associated with MDR *K. pneumoniae* infections in Brazil and other regions^10,11^.

Our results showed that human isolates grouped with animal and/or environment samples from different isolation years and sources, sharing the same aminoglycosides resistance genes and phenotypic profile. *Klebsiella* species are ubiquitously found in nature, including plants, animals, and humans. Studies about *K. pneumoniae* in veterinary medicine remain scarce, and the risk of human infection following contact with water, animals, or food is not well understood^12^. However, a recent study on the molecular-genetic characteristics of *Klebsiella spp*. isolates from animal and food sources reported high genetic diversity, with 62 STs identified among 67 *K. pneumoniae sensu stricto* isolates^13^. Moreover, animal and environmental isolates often show MDR phenotypes.

The effectiveness of aminoglycosides is compromised by the evolution of bacterial resistance mechanisms^14^. The most widespread resistance mechanism to aminoglycosides is their inactivation by AMEs and enzymatic modification by methyltransferases (MTases)^15^. Common aminoglycosides-resistance identified in our clinical KP strains include *rmtA* and *rmtB* (encoding 16S rRNA methylases), as well as *aac(3’)-Ia*, *aac(6’)-Ib*, *aac(3’)-IIa*, *aac(3’)-IId*, *ant(2’)-Ia*, *ant(3’)-Ia*, and *aph(3’)-Ia* (encoding aminoglycoside-modifying enzymes). Furthermore, various aminoglycosides-modifying genes have been reported in nonfermenting Gram-negative bacteria^16^. High-levels of aminoglycoside resistance are primarily caused by the production of acquired 16S-RMTase in pathogenic Gram-negative bacteria. These mechanisms confer high-level resistance (MIC >256 μg/mL) to all clinically relevant aminoglycosides, such as amikacin and gentamicin. Brazilian isolates harboring 16S-RMTase genes (*rmtB* and *rmtG*) showed high-level amikacin resistance (MIC > 128 μg/mL). In our study, high levels of gentamicin resistance (> 80% of human isolates) and moderate levels of amikacin resistance (~50% of human isolates) were observed based on MIC values.

Additionally, all isolates of this study carried AMEs, with *aph (3’)-Ia* being the most frequent gene, showing MIC ranges of 8–256 μg/mL for amikacin and gentamicin. Our results corroborate those found in previous studies that reported *aph (3’)-Ia* as the most predominant AME among *K. pneumoniae* clinical isolates, which exhibited high levels of gentamicin resistance. Similarly, an European study reported aminoglycoside resistance in *K. pneumoniae* isolates, as well as frequent production of AMEs such as *aac(6’)-Ib*, and *aac (3)-IIa* ^17^. Notably, AMEs genes are often located on mobile genetic elements—including as plasmids, integrons, and transposons—facilitating their dissemination across One Health compartments.

Molecular typing revealed diverse patterns among *K. pneumoniae* strains. Although ST258 and its derivative ST512 are dominant carbapenem-resistant *K. pneumoniae* (CRKP) lineages in the Americas and southern Europe, they are relatively rare in other regions of the world^18^. ST11 is the predominant MLST associated with high-risk CRKP strains in North America, Europe, and Asia. Likewise, since 2011, several studies have described the predominance of ST258 in *K. pneumoniae* isolates from Brazilian hospitals^13^. The detection of strains belonging to the MDR *K. pneumoniae* clonal complex 258, including single-locus variants ST11 and ST437, is of significant concern. The spread of MDR strains in Brazil has been particularly associated with ST258, ST11, and ST340.

A phylogenetic analysis was performed to understand the evolutionary relationship of these *K. pneumoniae* isolates. The isolates grouped heterogeneously, regardless of isolation site, source, or even ST. Usually, phylogenetic analyses of *K. pneumoniae* isolates reflect clustering according to MLST type. However, recent studies have reported that isolates with different STs may also cluster together across environmental and human sources^18^. Our analysis identified eight pairs of isolates from human, animal, and/or environmental origins that grouped closely, suggesting potential transmission between them. However, SNP analysis showed that five of these pairs shared more than 2,000 SNPs, indicating that their clustering may result from the absence of intermediate genomes rather than direct transmission. Two pairs shared between 500 and 700 SNPs, suggesting that transmission cannot be entirely ruled out, although it is less likely. Conversely, the pair Kp04 and Kp57, both isolated in 2011, shared only 60 SNPs, suggesting that the same clone may be circulating in both the environment (urban river) and humans, potentially representing a transmission source. Pérez-Vásquez *et al*.^19^ described a substitution rate of 12–30 SNPs per genome per year for ST11 *K. pneumoniae* isolates in a molecular clock analysis; in this Brazilian clone, three years and 60 SNP separate the isolations. It is important to note that these genomic analyses were performed using draft genomes. The synteny analysis between KP04 and KP57 showed high overall conservation but also revealed three genomic gaps, several translocations, and one inversion, mainly in regions associated with mobile genetic elements. These findings highlight the genomic plasticity of *K. pneumoniae* and the role of horizontal gene transfer in its diversification.

These findings reinforce that animals and the environment may be potential reservoirs of antimicrobial-resistant bacteria and clinically significant resistance genes. Our results support the hypothesis of transmission between humans and animals based on the presence of identical healthcare-associated clones and other highly virulent human strains in animals^20^. Moreover, the presence of multiple plasmid types may facilitate the horizontal transfer of resistance genes, contributing to the emergence and dissemination of MDR *K. pneumoniae* strains. Further research is needed to fully understand the mechanisms of plasmid-mediated antibiotic resistance and to develop effective strategies to control their spread. In this context, our correlation analysis revealed a strong positive association between the presence of the 16S rRNA methyltransferase genes (*rmtB* and *rmtG*) and the AME gene *aph(3’)-Ia* with elevated MIC values for amikacin and gentamicin. This observation is consistent with previous reports showing that these genes confer extremely high-level aminoglycoside resistance in *K. pneumoniae* and other Enterobacterales^16^. These findings underscore the functional relevance of these determinants across isolates from human, animal, and environmental sources, emphasizing their contribution to the dissemination of clinically significant resistance traits.

A limitation of our study was the restricted availability of isolates meeting the strict inclusion criteria, specifically *K. pneumoniae* strains from human, animal, and environmental sources with both phenotypic and genotypic aminoglycoside resistance data. However, we evaluated the molecular and phenotypic characterization of aminoglycoside resistance in *K. pneumoniae* isolates from Brazil using different international clones for which aminoglycoside resistance data were available.

## CONCLUSION

In this study, ST11, ST258, and ST340 were the predominant sequence types. The aminoglycoside resistance among these isolates was marked by a high frequency of resistance genes and elevated MIC values, indicating the widespread presence of aminoglycoside resistance determinants across human, animal, and environmental sources. Notably, genes such as *aph(3’)-Ia*, *aac(6’)-Ib*, and *aac(3)* were widely distributed and appear capable of being exchanged between human and non-human isolates. These findings underscore the role of animals and the environment as plausible reservoirs for these resistant strains, emphasizing the broad dissemination and public health relevance of aminoglycoside resistance in diverse settings.

## Data Availability

The complete anonymized dataset supporting the findings of this study is available at https://doi.org/10.48331/SCIELODATA.0TA4MI
