# Supplementary material for: Characterization of aminoglycoside resistance in multidrug-resistant Klebsiella pneumoniae isolates
Source: Rev Inst Med Trop Sao Paulo. 2025 Oct 3;67:e62. doi: 10.1590/S1678-9946202567062 (PMC12499518; doi:10.1590/S1678-9946202567062)
Supplement: Supplementary file 1 [file 1678-9946-rimtsp-67-S1678-9946202567062-suppl01.pdf]

REVISTA DO INSTITUTO DE MEDICINA TROPICAL DE SÃO PAULO

JOURNAL OF THE SÃO PAULO INSTITUTE OF TROPICAL MEDICINE

SUPPLEMENTARY MATERIAL

https://doi.org/10.48331/SCIELODATA.0TA4MI

Characterization of aminoglycoside resistance in multidrug-resistant *Klebsiella pneumoniae* isolates

Saidy Vásconez Noguera [1,2,3], Ana Paula Marchi [1,3], Marina Farrel Côrtes [1,3], Nazareno Scaccia [1,3,4], Roberta Cristina Ruedas Martins [1], Maura Salaroli de Oliveira [3,4,5], Flavia Rossi [6], Anna Sara Levin [3,4,6], Silvia Figueiredo Costa [1,3,4], Lauro Vieira Perdigão Neto [1,3]

[1]Universidade de São Paulo, Faculdade de Medicina, Instituto de Medicina Tropical de São Paulo, São Paulo, São Paulo, Brazil

[2]Pontificia Universidad Católica del Ecuador, Facultad de Medicina, Quito, Pichincha, Ecuador

[3]Universidade de São Paulo, Faculdade de Medicina, Departamento de Moléstias Infecciosas e Parasitárias, São Paulo, São Paulo, Brazil

[4]Centres for Antimicrobial Optimisation Network, São Paulo, São Paulo, Brazil

[5]Universidade de São Paulo, Faculdade de Medicina, Hospital das Clínicas, São Paulo, São Paulo, Brazil

[6]Universidade de São Paulo, Faculdade de Medicina, Hospital das Clínicas, Divisão de Laboratório Central Serviço de Microbiologia Clínica, São Paulo, São Paulo, Brazil

Correspondence to: Saidy Vásconez Noguera
Pontificia Universidad Católica del Ecuador, Facultad de Medicina, Av. 12 de Octubre, Quito, Pichincha, Ecuador
Tel: +593 0959840788

E-mail: svasconezn96@gmail.com

Received: 21 February 2025

Accepted: 31 July 2025

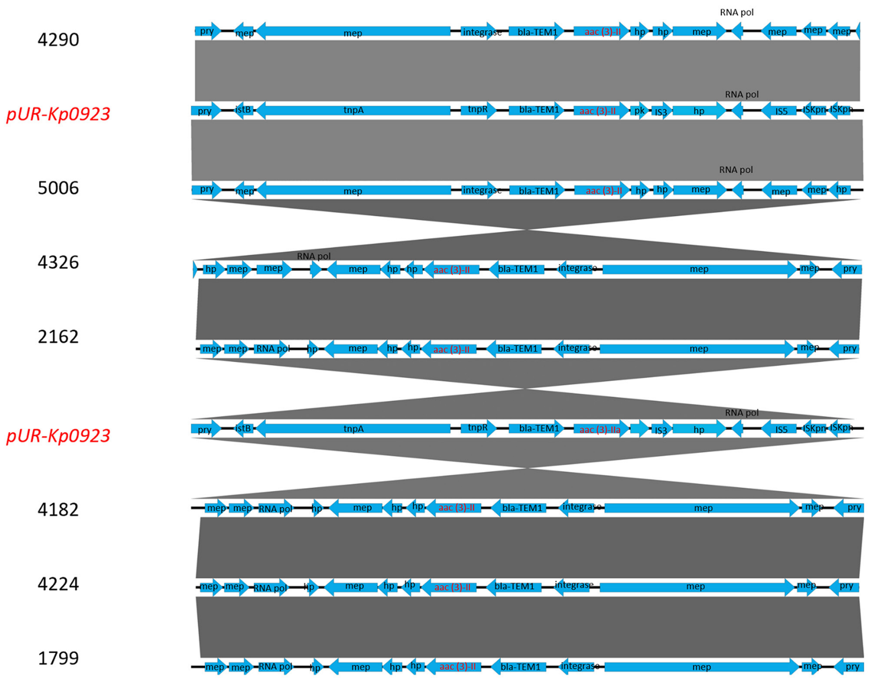

Supplementary Figure S1 - Schematic drawing showing genetic elements surrounding the aac (3´)-II gene in the plasmids described in this study. Arrows are proportional to the gene length and follow the transcription direction.

This is an open-access article distributed under the terms of the Creative Commons Attribution License.

Supplementary Table S1 - Sequence types, betalactamases, aminoglycoside resistance genes and antimicrobial susceptibility testing of 70 isolates of *K. pneumoniae*

Study Group	NCBI Accession number	ID number	Year	Source	Country	MLST	Antimicrobial Susceptibility Testing MIC (g/ml)				ESBL gene	Aminoglycoside resistance genes
							MEM	COL	GEN	AK		
E	SRR5713913	KP01	1980	Horse	France	23	S	S	R	R	*SHV-11*	*aadA, aph3-Ia*
	GCA_013180385.1	KP02	2005	blood	Brazil	11	S	S	R	R	*blaTEM, blaCTX-M2*	*aadA1, aadA2,aac(3)-Iia, aph(3)-Ia, aph(3)-Via*
	SRR1561227	KP03	2009	Human	Guatemala	11	S	S	R	R	*blaCTX-M-15,TEM-1D, SHV-11*	*aac(3)-IId, aac(3)-VIa (homolog), aadA2, aadA, aph3-Ia, armA*
	GCA_001968755.2	KP04	2011	Urban river	Brazil	437	R	R	S	S	*blaCTX-M15, blaKPC-2*	*aph(3)-Ia*
	GCA_001884385.1	KP05	2013	Hospital room swab	Canadá	278	R	S	S	S	*NDM-7, CTX-M-15, TEM-1, SHV-27*	-
	EuSCAPE_ES085	KP06	2013	Blood	Spain	11	R	S	R	R	*blaKPC-2, SHV-11*	*aac(3)-IV, aadA2, aadA, aph(3')-IIa, aph(4)-Ia*
	EuSCAPE_DE024	KP07	2013	Urine	Germany	147	R	S	S	S	*blaKPC-2, SHV-11*	
	EuSCAPE_RO115	KP08	2013	Lower respiratory tract secrection	Romania	101	R	S	R	R	*blaCTX-M-15, OXA-48*	*aac(3)-IIa, aac(6')-Ib', aadA (homolog)*
	EuSCAPE_DE051	KP09	2014	Blood	Germany	258	R	NA	R	I	*TEM-1D, SHV-11, blaKPC-2*	*aac(6')-Ib', aadA2, aph3-Ia*
	EuSCAPE_DE010	KP10	2014	Lower respiratory tract secrection	Germany	101	R	R	R	R	*OXA-1, OXA-9, TEM-1D, SHV-1, blaCTX-M-15, OXA-48*	*aac(3)-IIa, aac(6')-Ib', aadA (homolog)*
	EuSCAPE_ES293	KP11	2014	Wound secretion	Spain	258	R	NA	R	R	*TEM-1D, SHV-11, blaKPC-3*	*aac(6')-Ib', aph3-Ia*
	EuSCAPE_UK070	KP12	2014	Urine	UK	13	R	S	S	S	*SHV-1, blaKPC-2*	-
	EuSCAPE_IL061	KP13	2014	Wound secretion	USA	13	S	S	R	R	*blaCTX-M-15, OXA-1, SHV-1*	*aac(3)-IIa, aac(6')-Ib-cr, aadA5, aph3-Ia*
	EuSCAPE_ES257	KP14	2014	Urine	Spain	278	S	S	R	R	*SHV-27*	*strA, strB*
	EuSCAPE_PT025	KP15	2014	Urine	Portugal	11	S	S	R	R	*DHA-1, OXA-1, SHV-11*	*aac(6')-Ib-cr, aph3-Ia*
	GCA_002251715.1	KP16	2014	Rectal swab	Brazil	437	R	R	S	R	*laSHV11, blaCTX-M15, blaTEM-1 and blaOXA-1, blaKPC-2*	*(aph(3')-Ia, ac(3)-IId and ac(6')Ib-cr),*
	GCA_003063065.1	KP17	2014	Cloacal swab	Germany	3128	NA	NA	R	NA	*blaSHV–2, blaSHV–1, fosA-like, sul1, dfrA12, tet(D),*	*aadA1, aadA2, aac(3)-like,*
	GCA_000805735.1	KP18	2014	Rectal swab	Brazil	323	R	NA	R	R	*blaNDM-1, blaTEM, blaSHV, blaCTX-M, qnrA*	*aadA, aac(3)-IIa, aac(6')-Iq*
	GCA_004127475.1	KP19	2014	Dog swab anal	China	147	S	NA	R	NA	*blaNDM-5*	*NA*
	G18000639	KP20	2015	Urine	Colombia	13	R	S	R	R	*blaCTX-M-15, OXA-1, SHV-1, blaKPC-3*	*aac(3)-IIa, strA, strB*
	NZ_CP026392.1	KP21	2016	Wastewater/ sludge	USA	252	R	S	S	S	*SHV-1, blaKPC-2*	-
	G18001008	KP22	2016	Urine	Colombia	340	R	NA	R	R	*OXA-1, SHV-11, blaCTX-M-15, blaKPC-2, NDM-1*	*aac(3)-IIa, aac(6')-Ib-cr, aadA16 (homolog), aadA2, strA, strB*
	CP025515.1	KP23	2016	Wastewater	Switzerland	147	R	NA	R	R	*ctx-M15, bla-NDM-9, blaOXA-9and blaTEM-1*	*aacA4, aadA1, aadA1_2 and aph(3)-VI*
	GCA_002934865.1	KP24	2016	Rectum pig	Cameroon	3794	S	S	R	S	*blaSHV-1, blaCTX-M-15, blaTEM-1B*	*aac(3)-IIa , aadA1, strA, strB,*
	GCA_003583155.1	KP25	2016	River water	Austria	11	I	S	S	NA	*blaCTX-M-15, , blaOXA-1, blaOXA48*	*, aph3-Ia, aadA2, aac(6')Ib-cr,*
	SRR5943226	KP26	2016	Pig	Thailand	1	S	R	R	R	*CMY-2, SHV-1, blaCTX-M-14*	*aac(3)-IIa, strA, strB*
	GCA_002934845.1	KP27	2016	Nasal swab Pig	Cameroon	14	S	S	S	S	*blaTEM–116, blaSHV–28, blaCTX-M-15*	*strA, strB,*
	GCA_002934815.1	KP28	2016	Hand swab human	Cameroon	307	S	S	R	R	*blaOXA–9, blaLEN–12, blaSHV–134, blaTEM–1A,*	*aac(6')-Ib, strA, strB, aac(3)-IId, aac(6')Ib-cr*, aadA1, aadA16,*
	G18001049	KP29	2017	Blood	Colombia	11	R	R	R	R	*OXA-1, TEM-104, SHV-11,blaCTX-M-15, blaKPC-3*	*aac(3)-IId, strA (homolog), strB (homolog)*
	SRR11486232	KP30	2017	Human	Argentina	307	R	S	R	R	*blaCTX-M-15, OXA-1, SHV-28, blaKPC-2*	*aac(3)-IIa, aac(3)-IV, aadA2, aadA, aph(4)-Ia, strA, strB*
	SRR10153258	KP31	2017	Human	Brazil	11	R	S	R	R	*blaNDM-1, blaCTX-M-15, OXA-9, TEM-1D, SHV-11*	*aac(6')-Ib', aadA, aph(3')-VI*
	SRR10581305	KP32	2018	Cat	brazil	273	S	S	R	R	*blaCTX-M-15, OXA-1, TEM-1D, SHV-11*	*aac(3)-IIa, aac(6')-Ib-cr, strA, strB*
	GCA_011078225.1	KP33	2018	Soil in chicken farm	China	789	R	R	R	NA	*blaNDM-5*	-
	GCA_016055535.1	KP34	2019	Blood	Brazil	258	R	R	R	R	*blaKPC-2*	*aph(3')-Ia*
	GCA_013303005.1	KP35	2019	Urine dog	Brazil	258	R	S	R	R	*blaKPC–2, blaCTX-M–15, blaLAP–2, blaOXA–1, blaSHV–11*	*aac(3)-IIa, aadA2, aph(3')-Ia*
I	QEFS00000000	KP36	2011	Tracheal secretion	Brazil	11	8 (R)	0,5 (S)	≤ 4 (S)	8 (I)	*blaCTX-M-2, blaKPC-2, blaTEM-1B*	*aac(3)-IIa, aadA1, aadA2, aph(3')-Ia, aph(3')-VIa*
	QEGC00000000	KP37	2011	Nervous tissue	Brazil	442	> 8 (R)	32 (R)	> 32 (R)	>256 (R)	*blaOXA-9, blaKPC-2, blaTEM-1A*	*aac(6')-Ib, aadA1, aadA2, aph(3')-Ia, rmtG, aac(6') Ib-cr*
	QEFT00000000	KP38	2011	Urine	Brazil	11	>4 (R)	32 (R)	64 (R)	32 (R)	-	*aadA1, aph(3')-VIa, aac(3)-Iia*
	QEFY00000000	KP39	2012	Tracheal secretion	Brazil	340	≤ 1 (S)	1 (S)	> 32 (R)	>256 (R)	*blaCTX-M-14, blaSHV-27, blaTEM-1B*	*aac(3)-IId, aadA2, aph(3')-Ia*
	QEFX00000000	KP40	2012	Bone marrow	Brazil	258	8 (R)	64 (R)	8 (S)	64 (R)	*blaCTX-M-15, blaKPC-2, blaOXA-1, blaTEM-1B*	*aac(3)-IId, aadA2, aph(3')-Ia, rmtB, strA, strB*
	MZZQ00000000	KP41	2013	Blood	Brazil	340	> 8 (R)	32 (R)	> 32 (R)	>256 (R)	*blaCTX-M-14, blaKPC-2, blaTEM-1B*	*aac(3)-IId, aadA2, aph(3')-VIa, strA, strB*
	NBSC00000000	KP42	2013	Urinary catheter	Brazil	340	≤ 1 (S)	32 (R)	16 (I)	4 (S)	*blaCTX-M-8, blaLEN9, blaOXA-9, blaTEM-1A*	*aac(3)-IId, aadA2, aph(3')-VIa, strA, strB*

Supplementary Table S1 - Sequence types, betalactamases, aminoglycoside resistance genes and antimicrobial susceptibility testing of 70 isolates of *K. pneumoniae* (cont.)

	QEFV00000000	KP43	2013	Tracheal secretion	Brazil	25	> 8 (R)	0,5 (S)	≤ 4 (S)	32 (R)	*blaCTX-M-15, blaKPC-2, blaSHV-11, blaTEM-1B*	*aac(6')-Ib, aadA1, aac(6')Ib-cr*
	MZZV00000000	KP44	2013	Blood	Brazil	11	> 8 (R)	12 (R)	≤ 4 (S)	32 (R)	*blaCTX-M-2, blaCTX-M-8, blaKPC-2, blaOXA-2, blaSHV-11*	*aac(3)-IIa, aacA4, aac(6')Ib-cr*
	MZZX00000000	KP45	2013	Blood	Brazil	11	> 8 (R)	48 (R)	8 (S)	128 (R)	*blaCTX-M-2, blaKPC-2, blaOXA-2, blaSHV-11, blaTEM-1B*	*aac(3)-IIa, aacA4, aadA2, aph(3')-Ia, aac(6')Ib-cr*
	NBSB00000000	KP46	2013	Blood	Brazil	11	> 8 (R)	64 (R)	8 (S)	128 (R)	*blaCTX-M-2, blaKPC-2, blaOXA-2, blaTEM-1B*	*aac(3)-IIa, aacA4, aadA2, aph(3')-Ia, aac(6')Ib-cr*
	MZZT00000000	KP47	2013	Blood catheter	Brazil	258	> 8 (R)	32 (R)	> 32 (R)	>256 (R)	*blaCTX-M-14, blaKPC-2, blaSHV-11, blaTEM-1B*	*aac(3)-IId, aadA2, aph(3')-Ia, rmtB, strA, strB*
	NBOT00000000	KP48	2013	Blood catheter	Brazil	11	2 (I)	16 (R)	16 (S)	>256 (R)	*blaCTX-M-2, blaKPC-2, blaOXA-2, blaSHV-11*	*aacA4, aadA1, aph(3')-Ia, aac(6')Ib-cr*
	QEFZ00000000	KP49	2012	Tracheal secretion	Brazil	234	> 8 (R)	0,125 (S)	8 (S)	128 (R)	*blaCTX-M-2, blaKPC-2, blaOXA-2, blaSHV-11, blaTEM-1B*	*aac(3)-IId, aadA2, rmtB, strA, strB*
	QOIC00000000	KP50	2013	Rectal swab	Brazil	11	4 (R)	64 (R)	32 (R)	>32 (R)	*blaKPC-2, blaTEM-1B, blaSHV-182*	*aadA2, aadB, aph(3')-Ia, strA, strB, aac(3)-Iia*
	MZZR00000000	KP51	2014	Blood	Brazil	11	> 8 (R)	12 (R)	≤ 4 (S)	64 (R)	*blaKPC-2, blaOXA-2, blaSHV-11, blaTEM-1B*	*aac(3)-IIa, aacA4, aadA2, aac(6')Ib-cr*
	QOUE00000000	KP52	2014	Ascitic fluid	Brazil	11	> 8 (R)	8 (R)	8 (S)	64 (R)	*blaCTX-M-2, blaKPC-2, blaOXA-2, blaOXA-9, blaSHV-36, blaTEM-1A*	*aac(3)-IIa, aacA4, aadA2, aph(3')-Ia, aac(6')Ib-cr*
	QOUG00000000	KP53	2014	Blood	Brazil	23	> 8 (R)	4 (R)	≤ 4 (S)	64 (R)	*blaCTX-M-2, blaKPC-2, blaLEN12, blaOXA-2, blaTEM-1B*	*aac(3)-IIa, aacA4, aac(6')Ib-cr*
	QOUF00000000	KP54	2014	Ascitic fluid	Brazil	11	> 8 (R)	16 (R)	> 32 (R)	256 (R)	*blaCTX-M-2, blaLEN12, blaOXA-2, blaTEM-1B*	*aac(3)-IIa, aac(6')-Iq, aacA4, aadA1, aph(3')-Ia, aac(6')Ib-cr*
I	QOVT00000000	KP55	2014	Intra-abdominal collection	Brazil	11	> 8 (R)	32 (R)	> 32 (R)	≤0,5 (S)	*blaCTX-M-15, blaKPC-2, blaSHV-11*	*aac(3)-IIa, aacA4, aadA2, aac(6')Ib-cr*
	QOVV00000000	KP56	2014	Colletion	Brazil	340	> 8 (R)	8 (R)	≤ 4 (S)	≤0,5 (S)	*blaKPC-2, blaLEN12, blaSHV-11*	*aadA2, aph(3')-Ia, aph(3')-Via*
	QOVU00000000	KP57	2014	Peritoneal fluid	Brazil	437	> 8 (R)	16 (R)	≤ 4 (S)	≤0,5 (S)	*blaCTX-M-15, blaKPC-2, blaOXA-1, blaSHV-11, blaTEM-1B*	*aadA2, aph(3')-Ia*
	MZZU00000000	KP58	2015	Blood	Brazil	340	> 8 (R)	16 (R)	≤ 4 (S)	64 (R)	*blaCTX-M-2, blaKPC-2, blaOXA-2, blaTEM-1B*	*aac(3)-IIa, aac(6')Ib-cr, aph(3')-Ia, strA, strB*
	QOIA00000000	KP59	2015	Blood	Brazil	11	>16 (R)	32 (R)	16 (I)	4 (S)	*blaKPC-2, blaTEM-1B, blaOXA-2, blaSHV-182*	*aadA2, aac(3)-Iia, aac(6')-Ib3, aac(6')Ib-cr*
	NBOU00000000	KP60	2015	Blood	Brazil	11	> 8 (R)	64 (R)	> 32 (R)	>256 (R)	*blaCTX-M-14, blaKPC-2, blaSHV-11, blaTEM-1B*	*aacA4, aac(3)-IIa, aadA2, aph(3')-Ia, aac(6')Ib-cr*
	MZZY00000000	KP61	2015	Blood	Brazil	258	> 8 (R)	12 (R)	> 32 (R)	4(S)	*blaCTX-M-15, blaKPC-2, blaOXA-1*	*aac(3)-IId, aadA2, rmtB, strA, strB*
	NBIT00000000	KP62	2015	Blood	Brazil	16	> 8 (R)	12 (R)	≤ 4 (S)	32 (R)	*blaCTX-M-15, blaOXA-1, blaTEM-1B*	*aac(6')Ib-cr, aadA2, aph(3')-Ia*
	NBSD00000000	KP63	2015	Blood	Brazil	101	> 8 (R)	24 (R)	≤ 4 (S)	≤0,5 (S)	*blaCTX-M-15, blaKPC-2, blaSHV-11*	*aac(3)-IId, aac(6')Ib-cr, aph(3')-Ia, strA, strB*
	MZZW00000000	KP64	2014	Blood catheter	Brazil	340	> 8 (R)	8 (R)	> 32 (R)	>256 (R)	*blaCTX-M-14, blaKPC-2, blaSHV-11, blaTEM-1B*	*aph(3')-Ia*
	MZZS00000000	KP65	2016	Blood	Brazil	258	2 (I)	32 (R)	32 (R)	64 (R)	*blaCTX-M-15, blaSHV-11, blaTEM-1B*	*aac(3)-IId, aadA2, rmtB, strA, strB*
	QORQ00000000	KP66	2016	Blood	Brazil	16	≥16 (R)	64 (R)	8 (S)	4 (S)	*blaCTX-M-15, blaOXA-1*	*aadA2, aph(3')-Ia, aac(6')Ib-cr*
	QOUA00000000	KP67	2017	Tracheal secretion	Brazil	258	≥16 (R)	64 (R)	> 32 (R)	>256 (R)	*blaKPC-2, blaTEM-1B, blaCTX-M-14, blaSHV-182*	*aadA2, aph(3')-Ia, strA, strB, aac(3)-Iid, rtmB*
	QOHV00000000	KP68	2017	Blood	Brazil	11	≥16 (R)	16 (R)	15 (I)	4 (S)	*blaKPC-2*	-
	QOHS00000000	KP69	2017	Urinary catheter	Brazil	258	≥16 (R)	32 (R)	> 32 (R)	256 (R)	*blaKPC-2, blaTEM-1B, blaCTX-M-14, blaSHV-182*	*aadA1, aph(3')-Via, aac(3)-Iia*
	QOTX00000000	KP70	2017	Blood	Brazil	11	≥16 (R)	16 (R)	8 (S)	64 (R)	*blaKPC-2, blaTEM-1B, blaOXA-2, blaSHV-182*	*aac(3)-Iia, aac(6')-Ib3, aac(6')Ib-cr*

E = external group; I = internal group; MLST = Multilocus sequence typing; MIC = minimal inhibitory concentration; MEM = meropenem; COL = colistin; GEN = gentamicin; AK = Amikacin; ESBL = Extended-Spectrum β-Lactamase; AMEs = Aminoglycoside-Modifying Enzymes (chromosomal aminoglycoside phosphotransferase gene, aph(3')-Iib; gene aac(6')- Streptomycin 3"- adenyltransferase; AAC = aminoglycoside acetyltransferase(3–2"); AAD = aminoglycoside adenyltransferase; aph = aminoglycoside phosphotransferase; Quinolone-resistance genes = acetyltransferase AAC(6')-Ib-cr); R = resistant; I = intermediate; S = susceptible; NA = Not Available.
